# Urban infestation by *Triatoma infestans* (Hemiptera: Reduviidae), an overlooked phenomena for Chagas disease in Argentina

**DOI:** 10.1590/0074-02760210056

**Published:** 2021-06-02

**Authors:** Yael Mariana Provecho, María del Pilar Fernández, Liliana Salvá, Sergio Meli, Florencia Cano, Paula Sartor, Ana Laura Carbajal-de-la-Fuente

**Affiliations:** 1 Universidad de Buenos Aires, Facultad de Ciencias Exactas y Naturales, Departamento de Ecología, Genética y Evolución, Ciudad Universitaria, Buenos Aires, Argentina; 2 Consejo Nacional de Investigaciones Científicas y Técnicas, Instituto de Ecología, Genética y Evolución de Buenos Aires, Ciudad Universitaria, Buenos Aires, Argentina; 3 Ministerio de Salud de la Nación, Dirección de Control de Enfermedades Transmitidas por Vectores, Buenos Aires, Argentina; 4 Washington State University, Paul G Allen School for Global Animal Health, Washington, United States; 5 Ministerio de Salud de San Juan, Programa de Control de Vectores, San Juan, Argentina; 6 Universidad Nacional del Nordeste, Facultad de Ciencias Exactas, Naturales y Agrimensura, Corrientes, Argentina; 7 Ministerio de Salud Pública del Chaco, Resistencia, Chaco, Argentina; 8 Consejo Nacional de Investigaciones Científicas y Técnicas, Buenos Aires, Argentina; 9 Ministerio de Salud de la Nación, Centro Nacional de Diagnóstico e Investigación en Endemo-epidemias/Administración Nacional de Laboratorios e Institutos de Salud Dr Carlos Malbrán, Buenos Aires, Argentina

**Keywords:** urban infestation, vector control, San Juan

## Abstract

Vector-borne transmission of Chagas disease in urban areas of Argentina has been an overlooked phenomena. We conducted the first comprehensive cross-sectional study of domestic infestation with *Triatoma infestans* and vector infection with *Trypanosoma cruzi* in a metropolitan area of San Juan, Argentina. Our results document the occurrence of *T. infestans* infected with *T. cruzi* in human sleeping quarters. In this urban setting, we also show that infestation was associated with construction materials, the presence of chickens, cats and a large number of dogs that can provide blood meals for the vector. Our findings reveal new challenges for vectorial control agencies.

Chagas disease in a complex and multidimensional phenomenon, caused by the parasite *Trypanosoma cruzi* (Chagas, 1909), is transmitted to humans and other mammals by blood-sucking insects called triatomines. They are widely distributed in the Americas from the Great Lakes of northern USA to southern Argentina and Chile. *Triatoma infestans* (Klug, 1834) is the most important vector in the southern cone countries of South America.^1^ Although its historical-geographical distribution has been strongly reduced, after the implementation of the Southern Cone Initiative, this species still persists in some regions of Argentina, Bolivia and Paraguay where it represents an important socio-environmental health problem.^1^ In Argentina, there are still provinces with risk of vectorial transmission, although epidemiological data are outdated and were obtained mainly from entomological evaluations carried out in rural areas.^2^ The current extension of infestation by *T. infestans* may be higher than previously recognized, as a recent study compiling different data sources beyond official entomological evaluations showed that this vector has been reported in 92% of the territory (22/24 jurisdictions) in the post-2000 period, although reports mainly occurred in the Dry Chaco and Monte eco-regions areas where *T. infestans* historically has been reported.^3^

Infestation in urban settings may represent hidden foci since elimination efforts are mostly concentrated in rural areas of endemic provinces.^4^ As a consequence of migrations, urbanization, modification of agricultural strategies and climate change, Chagas disease has trespassed the Latin American rural matrix that gave its identity for decades into urban areas.^5^ Urbanization may create conditions to propagate vectorial transmission of disease given the great density of people living in close proximity with domestic and peridomestic animals.^6^ However, this phenomenon has been understudied and overlooked by vector agencies.^5^

This study was conceived as part of a rapid intervention work coordinated with national and provincial health agencies to suppress vector infestation and *T. cruzi* transmission in a large metropolitan area (the city of San Juan) from Argentina. *T. infestans* is the main domestic vector that colonizes human dwellings mainly in rural areas of Argentina,^3^ but historical records have also shown *T. infestans* infestations in this metro area.^7^ ^,^ ^8^ Experienced technical personnel of the San Juan Vector Control Program conducted an entomological survey on 2,427 houses from San Juan city and contiguous urban areas, during 2016. An infestation rate between 2-20% was reported. A total of 766 reports of *T. infestans* presence in domiciles were notified by householders during 2017. Additionally, between 2016 and 2020, acute vectorial transmissions of *T. cruzi* to humans were confirmed by provincial health authorities. Herein, we conduct the first cross-sectional assessment of domestic infestation with *T. infestans* and vector infection with *T. cruzi* in a metro area in Argentina to cast light into the eco-epidemiology of Chagas disease in this new urban context.

The study was conducted during June-December 2017 in the Rawson Department, a contiguous urban area of San Juan, Argentina, which encompass 29,520 houses in 300 km^2^ and 114,386 inhabitants (Fig. 1A-D).^9^ We selected 75 blocks (1 ha each) with approximately 35 houses each, totaling 2,612 houses. The selected blocks were representative of all types of houses in the area. All houses were visited, georeferenced, and 1,784 (68%) were inspected for the presence of triatomine vectors, after obtaining oral consent from an adult dweller. The remaining houses could not be inspected because they were closed (n = 581, 22%), uninhabited (n = 26, 1%) or refused to participate in the study (n = 221, 8%). Triatomine collections were conducted by trained personnel from the Provincial and National Vector Control Program using active search. Domestic infestation refers to the occurrence of at least one live *T. infestans* in the domicile (i.e., an independent structure used as human sleeping quarters with internal bathroom, kitchen and living room) or any peridomestic structure of the house, while intra-domiciliary infestation refers only to findings of *T. infestans* in domiciles. Immediately after the entomological survey, infested and adjacent houses were sprayed with standard doses (5%) of flowable beta-cypermethrin (Sipertrin, Chemotecnica, Argentina). This protocol has been review by Comité de Ética en Investigación, Ministerio de Salud Pública de San Juan (Exp. No. 800-006169/19). All caught specimens were identified taxonomically following Lent & Wygodzinsky,^10^ and counted according to species and stage. *T. cruzi* infection was determined in a random sample of 80% of all collected -third to fifth instar- nymphs and adult bugs, using direct observation under optical microscope (40x).^11^

**Fig. 1:**
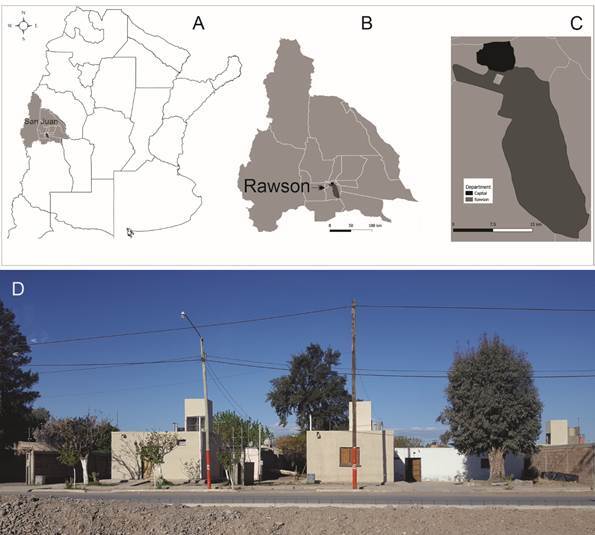
location of the study area in Argentina; (A) Location of San Juan province; (B) Rawson Department in San Juan Province; (C) Metropolitan area of Rawson Department (gray) with study area (white rectangle) showing the proximity to the Capital Department (black); (D) Urban houses with access to the power grid, natural gas, asphalted roads, drinking water, sewage and trash disposal.

Bivariate risk factor analysis for domestic and intra-domiciliary infestation was carried out via Firth penalized logistic regression implemented in R 3.6.3 (R Core Team 2020) using the package *logistf*, to account for small sample bias. The explanatory variables included those collected during a household survey delivered to an adult dweller or by direct observation during the vector survey: the number of dwellers, if the construction materials of walls and roofs could provide refuge for triatomines (e.g., mud or unplastered walls, thatched roofs, etc.), and the presence and number of non-human hosts (dogs, cats or chicken). The construction materials were only recorded for the domiciles. Multiple Firth penalized logistic regression models were conducted using a multi-model inference approach to account for model selection uncertainty, using the package MuMin.^12^ Models were ranked according to their Akaike Information Criterion adjusted for small sample sizes (AICc), and model averaging was conducted including those models within AICc < 2 from the best model. Multicollinearity was assessed by estimating the variance inflation factor.

A total of 958 *T. infestans* adults and nymphs were collected. At least one *T. infestans* was found in 7.0% (125/1,784) of the inspected houses. Infestation rates were similar in domiciles (3.6%, 63/1,784) and peridomestic sites (3.8%, 67/1,784) (χ^2^ = 3.14, df = 1, P = 0.08), but only five houses were infested in both. Of the 760 triatomines tested, 7.6% [95% confidence interval (CI) = 6.0-9.7%] were found infected with *T. cruzi* and came from 17 houses (1.0%, 17/1,784). The prevalence of *T. cruzi* infection among domiciliary (9.2%; 95% CI = 7.4-11.5%) and peridomestic (5.8%, 95% CI = 4.3-7.7 %) bugs was not significantly different (χ^2^ = 0.84, df = 1, P = 0.15) (Table I). Although no evident spatial pattern was observed, most of the blocks (64%) had at least one infested house (Fig. 2).

**TABLE I:** Prevalence of *Trypanosoma cruzi* infection *in Triatoma infestans* collected in houses from metropolitan area of San Juan, June-December 2017. The infected bugs came from 17 houses.

Infested houses	Stage	Nº of *T. infestans* examined by OM	Nº of infected (%)
Intradomicile	Third-fifth	134	12 (8.9)
	Adult	138	13 (9.4)
Peridomicile	Third-fifth	349	18 (5.2)
	Adult	217	15 (6.9)
Total		760	58 (7.6)

OM: optical microscopy (40x).

**Fig. 2:**
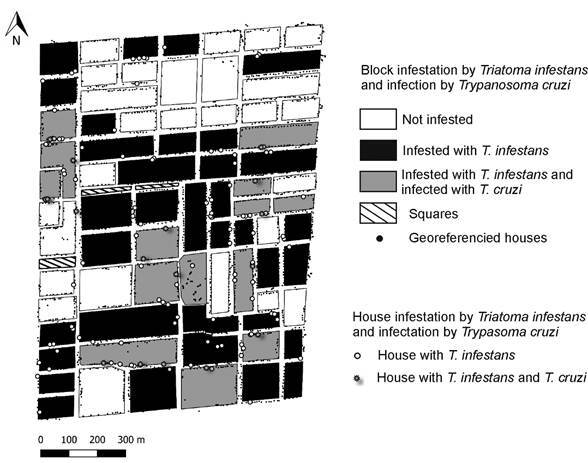
map showing the 1,784 inspected houses (black dots) georeferenced in the 75 blocks selected as the study area, and the location of houses infested with *Triatoma infestans* (white circles) and the occurrence of vectors infected with *Trypanosoma cruzi* (gray star) in the Rawson Department, San Juan province, Argentina, 2017.

Bivariate risk factor analysis showed that domestic infestation (in any site) was positively associated with the presence of cats and chickens (Table II). When ranking all models by AICc, the full model containing all variables was the best model and the remaining models had a delta AICc > 2. The full model confirmed the association of cats and chickens with house infestation and revealed the higher risk of infestation in houses with three or more dogs (Table III). Intra-domiciliary infestation was also associated with the presence of cats in bivariate analysis, and if the walls and/or roofs were built with materials that could offer refuge for triatomines (Table II). Three competing models were identified (AICc < 2 from the best model), and although the relative importance of the construction materials was 1 (i.e., present in the three models averaged), only the presence of at least one cat remained statistically significant (Table III).

**TABLE II:** Prevalence of domestic and intra-domiciliary infestation with *Triatoma infestans* and unadjusted odds ratio (OR) and 95% confidence interval (CI) obtained from bivariate. Firth penalized logistic regressions, for variables associated with the infestation by *T. infestans* in urban domiciles in the Rawson Department, San Juan, Argentina, June-December 2017.

Variable		Domestic infestation^*^	OR (CI)	Intra-domiciliary infestation^**^	OR (CI)
	N	% (N inspected)		% (N inspected)	
People per house					
	1-2	7.0 (673)	1	3.6 (673)	1
	3-5	6.7 (856)	0.9 (0.6-1.4)	3.0 (856)	0.8 (0.5-1.5)
	≥ 6	8.9 (213)	1.3 (0.8-2.3)	5.2 (213)	1.5 (0.7-3.1)
No. of dogs					
	0	7.7 (104)	1	2.9 (104)	1
	1	5.5 (454)	0.7 (0.3-1.5)	3.1 (454)	0.9 (0.3-3.1)
	2	8.0 (325)	1.0 (0.4-2.2)	4.6 (325)	1.5 (0.4-4.7)
	≥ 3	13.7 (285)	1.8 (0.8-4.0)	4.2 (285)	1.3 (0.4-4.4)
Cats					
	No	6.9 (823)	1	2.7 (823)	1
	Yes	11.9 (345)	1.8 (1.2-2.8)^***^	6.4 (345)	2.5 (1.4-4.5)^***^
Chickens					
	No	7.3 (1130)	1	3.7 (1130)	1
	Yes	42.1 (38)	9.3 (4.7-18.3)^***^	5.3 (38)	1.8 (0.5-6.6)
Wall and roof with materials that could offer refuge for triatomines					
Wall					
	No	-	-	2.0 (1274)	1
	Yes	-	-	7.8 (463)	4.0 (2.4-6.7)^***^
Roof					
	No	-	-	7.3 (629)	1
	Yes	-	-	1.4 (1109)	5.3 (2.9-9.3)^***^

*: domestic infestation refers to the occurrence of a live *T. infestans* in any domicile or peridomestic structure; **: intra-domiciliary infestation refers only to the presence of the vector inside the domicile; ***: p ˂ 0.05; N: number.

**TABLE III:** Adjusted odds ratio (ORadj) and 95% confidence interval (CI) obtained from the multiple Firth penalized logistic regressions for domestic and intra-domiciliary infestation. Using a multi-model inference approach, one model (the full model) was identified as the best model for domestic infestation, while three competing models (delta AICc < 2) were averaged for intra-domiciliary infestation. Relative importance of a variable refers to the relative presence of the variable in the competing models (1 = present in all three models; 0 = absent in all three models).

	Domestic infestation^*^	Intra-domiciliary infestation^**^	
Variable	ORadj (CI)	Relative importance	ORadj (CI)
Number of householders		0	
1-2	1		-
3-5	0.68 (0.42 - 1.1)		-
≥ 6	0.89 (0.47 - 1.67)		-
No. of dogs		0.7	
0	1		1
1	1.14 (0.5 - 2.86)		1.14 (0.34 - 3.81)
2	1.65 (0.73 - 4.15)		1.72 (0.52 - 5.7)
≥ 3	2.68 (1.22 - 6.59)^***^		1.36 (0.4 - 4.66)
Cats		1	
No	1		1
Yes	1.87 (1.19 - 2.9)^***^		2.41 (1.32 - 4.4)^***^
Chickens		0.2	
No	1		1
Yes	8.77 (4.28 - 17.79)^***^		1.16 (0.29 - 4.55)
Wall and roof with materials that could offer refuge for triatomines			
Wall		1	
No	-		1
Yes	-		0.24 (0.01 - 6.22)
Roof		1	
No	-		1
Yes	-		1.06 (0.04 - 26.56)

*: domestic infestation refers to the occurrence of a live *T. infestans* in any domicile or peridomestic structure; **: intra-domiciliary infestation refers only to the presence of the vector inside the domicile; ***: p < 0.05.

This study confirms the occurrence of domestic infestation with *T. infestans* infected with *T. cruzi* in a metropolitan area (> 500,000 inhabitants) in the province of San Juan, Argentina. Although a governmental plan to improve the quality of houses in the area was implemented in the Rawson Department during the last decade,^13^ our results show that intra-domiciliary infestation was associated with construction materials that could provide refuge to triatomines (e.g., if they had cracks in the walls). Similarly to other areas from Argentina, domestic infestation is associated with the presence of chickens, cats and a large number of dogs that can provide blood meals for the vector. A serological survey on dogs (n = 130) from the Rawson Department showed that 10% (n = 10) were reactive for *T. cruzi* [Sartor et al. (unpublished data)]. The role of cats and dogs is particularly important as infection sources for the vector.^14^ ^,^ ^15^ Given the proximity between the houses in this urban environment, the mobility and habits of these hosts might contribute to the dispersion of the parasite in the area. Although birds are refractory to *T. cruzi* infection, they can maintain triatomine colonies. Previous studies on pigeon lofts located in the metropolitan area indicate that they could be a continuous source of infestation and dispersion.^8^

In the urban context, the peridomicile is markedly different from the rural one. Although our results show that there are simple chicken coops and dog kennels, we did not find the large peridomiciliary structures such as goat corrals, traditional of rural areas with high abundance and infestation with *T. infestans*.^16^ Application of insecticide is carried out following the attack methodology in rural areas that present an infestation index < 5% or selective methodology when the index is > 5%.^4^ In urban areas, the scenario differs from rural settings by an increased population density is high (the last 2010 census indicates that in San Juan and its surroundings it is > 500,000 inhabitants) and house proximity, but also by the heterogeneity in construction materials (i.e., adobe, brick, blocks) and construction types (i.e., single-story homes or multi-story buildings). Thus, the vector indices thresholds for control may differ between settings, as well as the strategy used (i.e., spray coverage and distance radius from detected foci). These findings highlight the urgent need to develop new guidelines for cost-effective insecticide spraying to control the vector populations in this new urban context. Moreover, sustained and continuous coordination between health teams, researchers from multiple disciplines, public policy makers and the communities is critical. As recently proposed by Sanmartino et al.^17^ we believe that encouraging new reflections including Information, Education, and Communication (IEC) initiatives in Chagas, have great potential for impact in the implementation of multidimensional programs of prevention and control adapted to the urban context where transmision vectorial persists.
